# Influence of silver nanoparticles on growth and health of broiler chickens after infection with *Campylobacter jejuni*

**DOI:** 10.1186/s12917-017-1323-x

**Published:** 2018-01-02

**Authors:** Krishna Prasad Vadalasetty, Charlotte Lauridsen, Ricarda Margarete Engberg, Radhika Vadalasetty, Marta Kutwin, André Chwalibog, Ewa Sawosz

**Affiliations:** 1 Department of Veterinary and Animal Sciences, University of Copenhagen, 1870 Frederiksberg, Denmark; 2 Department of Animal Science, Aarhus University, 8830 Tjele, Denmark; 3 Department of Animal Nutrition and Biotechnology, Warsaw University of Life Sciences, 02-786 Warsaw, Poland

**Keywords:** Broiler chickens, Silver nanoparticles, Microflora, Immunoglobulins, Gene expression

## Abstract

**Background:**

Silver nanoparticles (AgNP) have gained much attention in recent years due to their biomedical applications, especially as antimicrobial agents. AgNP may be used in poultry production as an alternative to the use of antibiotic growth promoter. However, little is known about the impact of oral administration of AgNP on the gut microbiota and the immune system. The aim of the present study was to investigate the effects of AgNP on growth, hematological and immunological profile as well as intestinal microbial composition in broilers challenged with *Campylobacter jejuni* (*C. jejuni*).

**Results:**

AgNP did not affect the intestinal microbial profile of birds. The body weight gain and the relative weights of bursa and spleen were reduced when supplemented with AgNP. There was no difference with respect to packed cell volume. However, the plasma concentrations of IgG and IgM were lower in birds receiving AgNP compared to the non-supplemented control group. The expression of *TNF-α* and *NF-kB* at mRNA level was significantly higher in birds receiving AgNP.

**Conclusions:**

The application of AgNP via the drinking water in the concentration of 50 ppm reduced broiler growth, impaired immune functions and had no antibacterial effect on different intestinal bacterial groups, which may limit the applicability of AgNP against *C. jejuni* in broiler chickens.

**Electronic supplementary material:**

The online version of this article (doi: 10.1186/s12917-017-1323-x) contains supplementary material, which is available to authorized users.

## Background

In broiler production, different kinds of antimicrobial agents are used for preventing and controlling diseases. Antimicrobials can affect the host intestinal flora, by reducing the colonization of intestinal bacteria, inhibiting the growth of pathogenic microorganisms, and enhancing the immune system, hence preventing diseases and improving animal performance [1–3]. However, the overuse of antimicrobial agents (antibiotics) promotes the emergence of antibiotic resistance in microorganisms [4], being harmful to animal and human health [5, 6]. For example, resistance to ciprofloxacin in *Camplylobacter jejuni* (*C. jejuni*) isolated from Danish broiler meat increased significantly from 0% in 2009 to 17% in 2010 [7]. The use of all antibiotics as growth promoter has been prohibited in the European Union since 2006 [2, 8]. Thus, there is a need to find alternatives to antibiotics in poultry production. When the conventional therapies, including antibiotics, anti-inflammatory agents, growth hormones, surgical interventions, and cytotoxic chemotherapies are ineffective in curing poultry infections, it is necessary to explore novel drug compounds. Nanoparticles have been emerging as one of the new treatment options, and their capability of penetrating normally intact physiologic barriers has reached a variety of molecular targets [9, 10].

Recent studies on antibacterial materials such as various natural (oils, acids), inorganic antimicrobial agents such as metals (Ag, Au, Cu) and metal oxides (ZnO, SiO_2_, Fe_2_O_3_, TiO_2_) have received increasing attention. Among metal nanoparticles, silver is one of the most promising components in several nanotechnology products. Currently, there are several consumer products containing various silver nanoparticles (AgNP) because of their antimicrobial properties [11–14]. AgNP have been shown to have a wide range of antibacterial activities against both Gram-positive and Gram-negative bacteria, including major foodborne pathogens [15–19]. At present, there is no study available on their antibacterial effect against *C. jejuni* being a leading cause of human gastroenteritis worldwide. It is mainly transmitted from contaminated chicken meat. *Campylobacter* infections are associated with the neurological disorder Guillain-Barre syndrome [20, 21]. A common feature of *C. jejuni* causes enterocolitis and is involved in acute inflammatory response that can lead to tissue damage and may be responsible for many of the clinical symptoms [22]. Furthermore, antimicrobial resistance was observed in *C. jejuni* and *Campylobacter coli* [23].

Although the antimicrobial effect of AgNP has been studied extensively, the mechanism of antibacterial activity specific to bacteriostatic or bactericidal activity remains unclear. Studies had shown that AgNP upon contact with water can release Ag + ions from their surface [24]. Free Ag + has a potent antimicrobial effect, which destroys microorganisms immediately by blocking the cellular respiration and disrupting the function of bacterial cell membranes. This occurs when Ag + binds to tissue proteins, causing structural changes in the bacterial cell membranes which, in turn, cause cell death. Essential protein complexes of the bacterial electron transport chains are located on the cell exterior and, therefore, are manageable for inactivation by reactive silver ions. Ag + also binds and denatures the bacterial DNA and RNA, thus inhibiting cell replication [25]. Recently, evidence has been obtained suggesting that silver nanoparticles may modulate the phosphotyrosine profile of putative bacterial peptides that could affect cellular signaling and, therefore, inhibit the growth of bacteria [26].

In recent years, several studies have been focused on anti-inflammatory therapy and on molecules which could block pro-inflammatory pathways. Nanoparticles of Ag and Au are considered anti-inflammatory agents or components of anti-inflammatory molecules [27, 28]. Moreover, in vivo studies with chicken embryos and quails showed that AgNP did not affect growth, development [29] and DNA oxidative damage to chicken embryos [30]. Results from toxicological assays have shown no in vitro cytotoxicity of AgNP (0.1, 0.5 and 1.0%) [17] but concentrations (2.5–50 μg/mL) of AgNP exert a cytotoxicity effect on human mesenchymal stem cells [31]. The most common health effects associated with chronic exposure to silver are a permanent grey or blue-grey discoloration of the skin (argyria). From the immunological perspective, it is known that phagocytosis of AgNP stimulates inflammatory signaling through the generation of reactive oxygen species (ROS) in macrophage cells, followed by the activated macrophage cell-induced secretion of TNF-α. The increase of TNF-α level causes damage to the cell membrane and apoptosis [32]. An inadvertent recognition of AgNP as a foreign particle by the immune cells may result in a multilevel immune response and finally lead to toxicity in the host. However, when the AgNP are recognized as self or an absence of immune recognition, then their ability to stimulate immune response may decide the fate of AgNP in the host. In vivo studies have demonstrated that nanoparticles are capable of promoting inflammation or suppressing immune functions [33–35]. The nanoparticle-induced inflammatory response may have an impact on immune defense, and the T-helper 1 (Th1)/T-helper 2 (Th2) balance [9, 36].

There are limited data regarding the effect of orally administered AgNP on the intestinal bacterial population and immune system of animals. In this study, we hypothesized that the antimicrobial properties of colloidal solutions of AgNP may affect the microbial population and immune responses upon challenge. The use of AgNP in poultry production may potentially function as an alternative to the use of antibiotic based growth promoters. The objective of this study was to investigate the effect of AgNP on the growth, the microbial profile of digestive tract and the immune status of broilers exposed to *C. jejuni* infection. In this study, we used hydrocolloid of AgNP because they exhibit high surface/volume ratio which may effectively enhance the bactericidal activity. In addition, chicks were used as an animal model for bacterial GI infection.

## Methods

### Experimental solution

The hydrocolloid of AgNP obtained from Nano-Tech (Warsaw, Poland) was produced by an electric non-explosive patented method (Polish Patent 3,883,399) from high purity metals (99.9999%) and high purity demineralized water. The concentration of the hydrocolloid was 50 mg/kg (50 ppm). The shape and size of nanoparticles were inspected by transmission electron microscopy (TEM), the particles had a crystal structure with an average size of 3.5 nm. The average surface area was 2.827 × 10^−13^ cm^2^ and the pH of the colloidal silver solution was 7.1 to 8.1 (data provided by Nano-Tech, Poland). Furthermore, more detailed information regarding the applied AgNP are given by Sawosz et al., 2011 [18].

### Experimental design

Ninety day-old male broiler chickens (Ross 308), obtained from a Danish commercial hatchery (DanHatch, Vrå, Denmark) were used. Upon arrival at the laboratory, the birds were wing-labeled, weighed and randomly distributed to two experimental groups: control, no AgNP, and provided with 50 ppm of AgNP in the drinking water. Furthermore, prior to the experiment antibacterial tests were done at in vitro level.

The chickens were housed in six individual isolators with 15 birds per isolator. The room temperature was 32 °C at the beginning of the experiment and was gradually decreased according to the demands of the growing chickens. The humidity was maintained during the experiment. Over the entire experimental period, both experimental groups were fed the same diet as described in Table 1. Drinking water was provided via nipple drinkers placed in the isolators. Birds were inspected every day and for every 2 days water and fed consumption were recorded. All birds had free access to food and water.

**Table 1:** Composition of the diet for broiler chickens (g/kg)

Ingredients	
Wheat	49.9
Maize	10.0
Rape seed (LL), grounded	4.00
Soybean meal (de-hulled, toasted)	29.4
Soybean oil	2.45
Calcium carbonate	0.90
Monocalcium phosphate	1.56
Sodium chloride	0.20
Natrium-bicarbonate	0.27
Lysine hydrochloride (100%)	0.25
DL-Methionine (100%)	0.35
Threonine (98%)	0.10
Vitamins and minerals (Vitfoss, Slut, 0.5%)	0.60
Total	100

At day 11, all chickens were weighed and cloacal swab samples of 5 chickens per isolator were taken and examined for the presence of *C. jejuni* colonies for initial observation. Subsequently, two birds were randomly selected from each treatment and killed, and then blood samples were collected for packed cell volume (PCV) determination before samples of liver tissue were collected for gene expression studies. Afterwards, the chickens were orally challenged with 0.5 mL of an overnight culture of *C. jejuni* (4 × 10^7^/ mL per bird). The infection strain (DVI-sc181) has been isolated from infected commercial broilers and provided by the Technical University of Denmark, National Veterinary Institute.

On days 15, 22 and 30, all chickens were weighed, and 5 chickens per isolator were randomly chosen. Blood samples were taken from the jugular vein and the chickens were immediately sacrificed by cervical dislocation. Liver, heart, spleen and bursa were collected, weighed and stored at -80 °C for further analysis. Ileum and caecum were collected to enumerate intestinal bacteria.

### Enumeration of *Campylobacter* and selected groups of intestinal bacteria

*C. jejuni* were enumerated in intestinal contents of ileum, caecum and faeces from 5 chickens per isolator at each sampling day. The samples (approximately 3 g) were homogenized, and serially diluted in 10-fold in phosphate buffered saline and plated on modified blood free charcoal cefoperazone deoxycholate agar base (Oxoid, CM0739). The plates were incubated with *C. jejuni* specific growth supplements at 42 °C for 48 h under microaerobic conditions (5% O_2_, 5% CO_2_, 5% H_2_, and 85% N_2_). Enterobacteria (*E. coli* and lactose negative enterobacteria) were enumerated on MacConkey agar (Merck, Darmstadt, Germany, 1.05465) incubated aerobically at 37 °C for 24 h as described by Engberg et al. [37]. Lactic acid bacteria (LAB) and *Clostridium perfringens* were counted respectively on De Man Rogosa Sharpe agar (Merck, 1.10660) incubated anaerobically at 37 °C for 48 h and tryptose sulphite cycloserine plates (TSC-Agar, Merck, 1.11072) incubated anaerobically at 37 °C for 24 h. Enterococci were counted on Slanetz and Bartely plates (Merck, 1.05289) after aerobic incubation at 37 °C for 48 h. The results are presented as microbial number (CFU/g) in ileal/caecal or faecal material. The detection limit was 10 ^2^ bacteria/g. During the experiment, water samples were collected from the drinkers in the isolators and the antimicrobial effect of AgNP on *C. jejuni* was investigated in in vitro using the plate count method.

### Hematology

Before cervical dislocation, blood was collected by puncture of the jugular vein in disposable sodium heparinized hematocrit capillary tubes (Camlamb Ltd., Cambridge, UK). The tubes were filled up to two-thirds and sealed with cristaseal (Hawksley, Sussex, UK). The percentage of packed cell volume (PCV) was measured by using a micro-hematocrit reader (Hearaeus Reader, Osterode, Germany).

### Concentrations of plasma immunoglobulins

From 5 chickens, blood samples were drawn from the jugularis vein and were collected in heparinized tubes. Blood samples were subsequently placed on ice, centrifuged at 2000 g for 10 min at 4 °C, and the plasma was stored at −20 °C until analysis of immunoglobulins. The concentrations of IgA, IgM and IgG were measured. Chicken plasma specific antibodies such as IgA (Bethyl laboratories, cat. no. E33–103), IgG (Bethyl laboratories, cat. no. E33–104) and IgM (Bethyl laboratories, cat. no. E33–102) concentrations were determined in diluted samples (1:100) by enzyme-linked immunosorbent assay (ELISA) using microtiter plates (NuncImmunoplate 96-well, cat. no. 446612) as per manufacturer’s ELISA quantitation kits (Bethyl Laboratories Inc., Montgomery, TX, USA).

Measurement of the content of specific chicken antibodies was done by indirect ELISA as follows: microtiter plate wells were coated with 100 μl of diluted coating antibody (1:200) to all wells and incubated for 2 h at room temperature (RT). After incubation, coated plates were washed with washing solution pH 8.0 (0.05 M Tris, 0.15 M NaCl, 10% Tween 20) to eliminate excess capture antibodies. Wells were incubated with 200 μl blocking buffer pH 8.0 (0.05 M Tris, 0.15 M NaCl, 1% BSA) on a shaker for 30 min at RT to block nonspecific protein binding and then washed 3 times with washing solution.

For the determination of immunoglobulins (IgG, IgM, or IgA), plasma samples were diluted and 100 μl of diluted plasma was added in triplicate. Plasma dilutions were 1:3000 for IgM, 1:1000 for IgA and 1:25,000 for IgG. Concentrations of IgG, IgM, and IgA in the standards were 6.25 mg/ml, 0.4 mg/ml, and 0.38 mg/ml respectively. Standards were diluted for IgG ranging from 200 ng/ml to 3.12 ng/ml, for IgM from 250 ng/ml to 3.9 ng/ml and for IgA from 1000 ng/ml to 15,625 ng/ml. The concentrations of immunoglobulins in test plasma samples were determined using these standard curves. The plates were incubated for 1 h at RT and were washed three times in washing solution. 100 μl of (horseradish-peroxidase) HRP detection anti-chicken IgM (A30-102P) was diluted 1:75,000 and for anti-chicken IgA (A30-103P) and IgG (A30-104P) were diluted 1:50,000 in conjugate diluent pH 8 (0.05 M Tris, 0.15 M NaCl, 10% Tween 20) was added to each well and the plates were allowed to incubate for 1 h at RT. After incubation, to remove unbound peroxidase-conjugates, each well was washed with washing solution for 3 times. 100 μl of (3,3,5,5-tetramethylbenzidine) TMB substrate was added to each well to determine bound peroxidase, and after incubation for 5–15 min in the dark at RT, 100 μl of 1 M H_2_SO_4_ was added to each well to stop the TMB reaction. The optical density was measured at 450 nm and expressed as ng of IgA, IgM and IgG per ml of plasma with a microplate reader. Using the mean absorbance value for each sample, determines the corresponding concentration of immunoglobulins in ng/ml from the standard curve.

### Gene expression of TNF-α and NF-kB

Liver tissue samples (approximately 30 mg) were homogenized in TRIzol® Reagent (Invitrogen, Carlsbad CA, USA, cat. no. 15596–018). Then, RNA clean-up was performed and total RNA was extracted according to the manufacturer’s protocol using SV Total RNA isolation system (Promega Corporation, Madison, WI, USA, cat. no. Z3105). Final RNA preparations were resuspended in RNase-free water and stored at -80 °C. The content of isolated RNA was quantified by UV-spectroscopy at 260 nm/280 nm with a NanoDrop® ND-1000 Spectrophotometer (Thermo Fisher Scientific, Waltham, MA, USA). Further quality was assessed by Agilent 2100 bioanalyser (Agilent RNA 6000 Nano kit, Waldbronn, Germany) and more than 6.5 RNA integrity number values were considered for cDNA preparation.

Two mg of total RNA was reverse-transcribed using the cDNA Reverse Transcription Kit (Promega, cat. no. A3500) in a G-storm PCR System. After this, real-time PCR was performed with complementary DNA and gene-specific primer pairs (TAG, Copenhagen A/S, Copenhagen, Denmark) mixed with LightCycler®480 SYBR Green I Master mix (Roche Applied Science, Penzberg, Germany) in a LightCycler® 480 real-time PCR system (Roche Applied Science). The cycling conditions included an initial heat step at 95 °C for 5 min, denaturation at 95 °C for 10 s, annealing temperatures described in Table 2 for each of the primers and product elongation at 72 °C for 20 s for 45 cycles. Following amplification, the melting curve was conducted on each sample to ensure that a single product was obtained using three-segment cycle of 95 °C for 5 s, 65 °C for 1 min and 95 °C no hold for continuous-acquisition mode with a heating rate of 0.11 °C/s and 5 acquisitions per 1 °C). Characterization of the product size was, furthermore, confirmed by agarose gel electrophoresis and sequencing (Bechman coulter genomics Takeley, UK). Results were quantified based on the relative expression of the *TNF-α* and *NF-kB* genes versus the housekeeping genes GAPDH and ACTB using advanced relative quantification (Efficiency method) by Light Cycler 480 Software release 1.50 SP4. The gene expression experiment was repeated 2 times chemically with consistent results and three replicates were used per sample each time.

**Table 2:** Genes and primers used in the study

RNA target gene	Primer sequences(5′–3′)	PCR Product		Gene bank Assess. No
		Annealing Temperature	Amplicon (bp)	
*GAPDH*				NM_204305.1
Forward	TGCTGCCCAGAACATCAT	61 °C	199	
Reverse	ATCAGCAGCAGCCTTCAC			
*ACTB*				NM_205518.1
Forward	GTCCACCTTCCAGCAGATGT	60 °C	169	
Reverse	ATAAAGCCATGCCAATCTCG			
*NF-kB*				M86930.1
Forward	TTGCTGCTGGAGTTGATGTC	60 °C	167	
Reverse	TTGCTGCTGGAGTTGATGTC			
*TNF-α*				NM_204267.1
Forward	TTCAGATGAGTTGCCCTTCC	59 °C	150	
Reverse	TCAGAGCATCAACGCAAAAG			

*GAPDH* Glyceraldehyde-3-phosphate dehydrogenase, *ACTB* Actin beta, *NF-kB* nuclear factor kappa B, and *TNF-α* Tumor necrosis factor alpha

### Statistical analysis

The data were analyzed using the GLM procedure of SAS (SAS Institute Inc., 2008) 9.2 version for windows considering the treatments (AgNP vs. non-supplemented control), the day of age (15, 22 and 30) and interactions between treatment and age. Tukey-Kramer significant different test was employed to test the separation of the means and differences were considered statistically significant when the *p* ≤ 0.05.

## Results

### Enumeration of bacteria

**Fig. 1:**
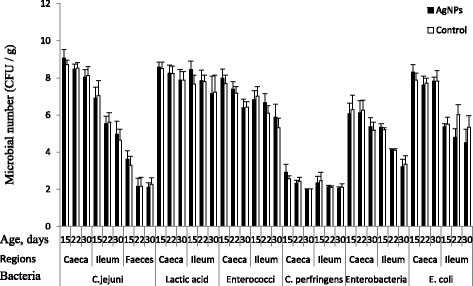
Influence of AgNP administration in broiler chickens after challenge with *C. jejuni* on microbial profile in caeca, ileum and feces. At 15, 22, and 30 days of age, samples (*n* = 30 for each time point) were used for microbial count. Error bars represent the mean and standard errors of 6 isolators each with 15 birds

There were no differences between birds receiving AgNP and the control group (Fig. 1) with respect to the numbers of *C. jejuni,* lactic acid bacteria*, Enterococci, Clostridium perfringens, Escherichia coli*, and Lactose negative *enterobacteria* in the contents of caecum, ileum and feces of the birds.

### The influence of AgNP on feed and water intake

AgNP had no effect on the average daily water intake (ADWI), average daily feed intake (ADFI) and feed conversion ratio (FCR = ADFI/ ADWG) (Table 3). The average daily weight gain (ADWG) was significantly lower in birds receiving AgNP compared to the control birds, in the periods 0–11 days (*p* = 0.007), 11–15 days (*p* = 0.002) 15–22 days (*p* = 0.003) but not in 22–30 days (*p* = 0.728). The average amount of AgNP which single chicken received in the drinking water was 8.26 mg/d (Additional file 1: Table S1).

**Table 3:** Effect of silver nanoparticles (AgNP) on daily feed and water intake, weight gain and feed conversion ratio of chickens infected with *C. jejuni*

Parameters	Mean		Pooled	Treatment*Age
(g/bird)	Control	AgNP	SE	*p*-value
ADWI				
Day 0–30	161	165	0.34	0.64
ADFI				
Day 0–30	97.0	102	0.17	0.16
ADWG				
Day 0–11	21.7^a^	18.7^b^		
Day 11–15	43.7^a^	38.6^b^		
Day 15–22	62.7^a^	57.4^b^		
Day 22–30	70.5	69.4		
Day 0–30	46.6	44.9	0.03	0.34
FCR				
Day 0–30	1.97	2.22	0.04	

*Indicates interaction

Values are mean of 6 isolators each with 15 birds

*SE* pooled standard error, *ADWI* Average daily water intake (g per bird), *ADFI* Average daily feed intake (g per bird), *ADWG* Average daily weight gain (g per bird) and *FCR* Feed conversion ratio

^a,b^ values within rows with different superscripts are significantly different at *p* <0.05

### The effect AgNP on chicken body weight and relative organ weights

The body weight of birds supplemented with AgNP was significantly lower than that of control bids (Table 4). Significant interactions between age and treatment were observed for the relative weight of bursa and spleen, where a lower relative bursa at age 15, and for spleen at age 30 were noticed for AgNP group. Relative organ weights of heart and liver were not different between the control birds and birds supplemented with AgNP.

**Table 4:** Effects of silver nanoparticles (AgNP) on cumulative body and relative organ weight gain (g/kg) in *C. jejuni* infected broiler chickens

Parameters (g/bird)	Mean		Pooled	Treatment*Age
	Control	AgNP	SE	p-value
AWG				
Age 15	380^a^	318^b^		
Age 22	788^a^	722^b^		
Age 30	1424^a^	1346^b^	0.305	0.013
Heart				
Age 15	0.71	0.70		
Age 22	0.62	0.66		
Age 30	0.47	0.50	9.17	0.340
Bursa				
Age 15	0.25 ^a^	0.20^b^		
Age 22	0.21	0.22		
Age 30	0.20	0.20	5.08	0.003
Spleen				
Age 15	0.09	0.11		
Age 22	0.07	0.07		
Age 30	0.07^a^	0.05^b^	2.31	<0.001
Liver				
Age 15	2.71	2.82		
Age 22	2.52	2.56		
Age 30	2.01	2.00	0.003	0.657
Abnormalities	At age 30, big gall bladder was found from 2 out of 15 birds in AgNP group			

*Indicates interaction

^a, b^ values within rows with different superscripts are significantly different at *p* <0.05. SE – pooled standard error, AWG – average body weight gain. The relative organ weights (weight of organ/100 g live body weight). Values are mean of 6 isolators each with 15 birds

### AgNP response on PCV

**Fig. 2:**
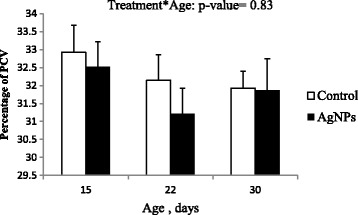
Effect of silver nanoparticles (AgNP) on packed cell volume levels in chickens infected with *C. jejuni*. Error bars represent the mean values and standard errors of 6 isolators each with 15 birds

At days 15, 22 and 30 of age no significant difference between control birds and birds receiving AgNP was observed with respect to blood PCV levels (Fig. 2). Likewise, no effect of age was observed.

### Plasma immunoglobulin concentrations

**Fig. 3:**
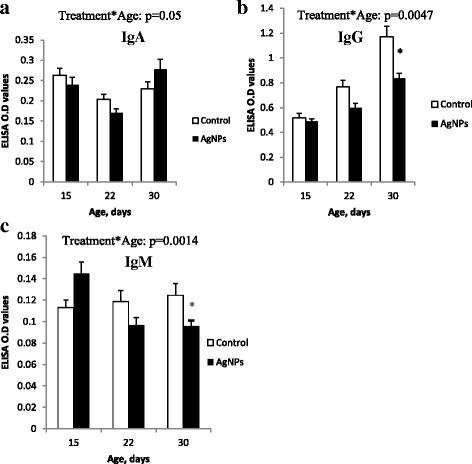
Concentration of immunoglobulins (IgA, IgG and IgM) in chickens infected with *C. jejuni* Samples were analyzed at days 15, 22 and 30 of age; **a**) IgA **b**) IgG and **c**) IgM. Error bars represent the mean values and standard errors of 6 isolators, each with 15 birds. Interactions between treatment and age effect significantly (*p* < 0.05). * Indicates significant difference between control and AgNP (*p* < 0.05)

We observed significant interactions between treatment and age for the concentrations of IgA, IgG and IgM in plasma (Fig. 3). At day 30, the plasma concentration of IgA was higher in birds supplemented with AgNP than in control birds, whereas the opposite effect was observed at age 15 and 22 days (*p* = 0.05, Fig. 3a). At day 30, the concentration of IgG in plasma was significantly lower in birds receiving AgNP as compared to the control birds (Fig. 3b). At age 15, plasma concentrations of IgM were higher in the AgNP group than in the control group (Fig. 3c), whereas at day 22 and 30, the concentrations of IgM were lower (*p* < 0.05).

### Gene expression of *NF-kB* and *TNF-α*

**Fig. 4:**
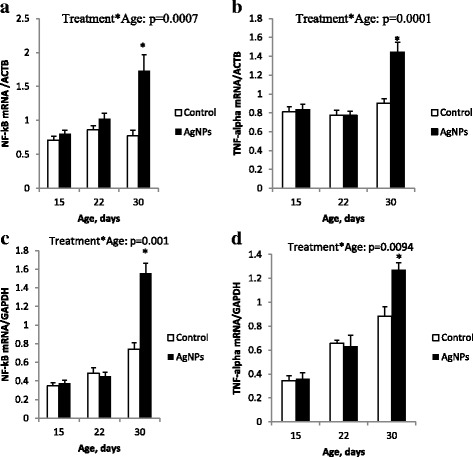
The expression of mRNA *NF-κB* and *TNF-α* normalized to the housekeeping genes *ACTB* and *GAPDH* in the liver tissue of chickens infected with *C. jejuni.* Samples were analyzed at days 15, 22 and 30 of age; **a**) *NF-κB* /*ACTB*, **b**) *TNF-α* /*ACTB*, **c**) *NF-κB* /*GAPDH* and **d**) *TNF-α* /*GAPDH*. Error bars represent the mean values and standard errors of 6 isolators, each with 15 birds. Interactions between treatment and age effect significantly (*p* < 0.05). * Indicates significant difference between control and AgNP (*p* < 0.05)

The influence of AgNP on the mRNA *NF-κB* and *TNF-α* expression in liver tissue of *C. jejuni* infected chickens is shown in Fig. 4. Interestingly, at day 30, the levels of *NF-κB* (Fig. 4a-c) and *TNF-α* expression (Fig. 4b-d) were increased (p < 0.05) in the group supplemented with AgNP compared to the control group.

## Discussion

In vitro antibacterial tests against *C. jejuni* showed a minimal inhibitory concentration of AgNP at the level of 40 and 50 ppm. AgNP have an effect on the target microorganism in the absence of chickens. In the present in vivo experiment, 50 ppm of AgNP was used because, prior to this experiment we carried out dose response studies to examine the antibacterial effect against *C. jejuni* of different concentrations of AgNP on chickens by chicken intestinal organ culture model (CIOC-model, Additional file 2: Table S2)*.* The minimum inhibitory and minimum bactericidal concentration of silver nanoparticles on *C. jejuni* was determined by using the microtiter plate method (Additional file 3: Figure S1), showed bactericidal effect with minimum concentration of 50 ppm. We have evaluated samples of ileum, caecum and feces because of the uniformity of the gut microbiota in these segments. The caecum has a slow digesta passage rate, allowing to harbor a complex microbiome that has considerable effects on host nutrition and health [38]. The small intestinal region of the ileum has received attention since it is the principal site of nutrient absorption and microflora [39]. Furthermore, we focused on test samples at various time points because the caecal and ileal microflora changes in relation to age and dietary treatments [38, 39].

However, unexpectedly, AgNP did not change the microbial profile of caecum, ileum and feces of examined chickens (Fig. 1). These results are consistent with in vivo experiments with the microbial profile of young quails receiving hydrocolloids of AgNP administered with 5, 15 and 25 ppm [29]. Moreover, another study demonstrated that supplying AgNP did not effectively rescue *Salmonella***-**mediated mortality in chickens but nanoscale silicate platelet (NSP) and its nanohybrid composite of AgNP/NSP effectively controlled the infection [40]. However, our present study was not similar with this study in terms of end point of examination, concentration of nanoparticles, bacterial infection time points and target bacterial species. The factors that could affect the present results might be the method of AgNP administration, although *C. jejuni* colonization in the intestinal tract depends on the number and diversity of environmental microorganisms, as well as influence of feedstuffs. The other possibilities could be the *C. jejuni* biofilm formation and adhesion to the host intestinal wall may provide protection against nanoparticles. The chicken intestinal mucus is able to attenuate *C. jejuni* virulence by inhibiting its ability to adhere and invade intestinal epithelial cells [41]. Furthermore, the antibacterial properties of AgNP could vanish inside the digestive tract environment of chickens, probably, due to AgNP agglomeration in the presence of low pH gastric acid. The obtained results may suggest that AgNP were gastro-sensitive, the stability and dispersion of AgNP in gastric acid is a critical factor for antibacterial activity. Once the particle enters a biological system, physical properties such as solubility, particle agglomeration, surface charge and particle-protein complex interactions might be different from those of the in vitro-measured properties. The loss of nanoparticles’ properties may be due to the low colloidal stability and the reduction of reactive surfaces may affect the efficacy for controlling pathogens in a solution [42].

The reduction in cumulative body weight and relative organ weights of the spleen and bursa of birds supplemented with AgNP might be due to the effect of AgNP blocking the intestinal absorption of actively transported sugars and amino acids, and decreased protein digestibility through the small intestine where mainly enzymatic activity is present. Another possible reason might be the fact that the birds undergo more cellular stress and excessive cellular interactions with AgNP. It appears that *C. jejuni* colonization may lead to weight loss in grower chickens. The present results are consistent with decreased body and organ weights in chickens treated with 25 ppm of AgNP at 42 days of age [43]. Moreover, the study of Asharani et al. [44] suggested that AgNP increase ROS production and interrupt ATP synthesis, leading to DNA damage and cell cycle arrest at G2/M phase. Park et al. [32] found that AgNP induce G1 phase arrest and a complete blockage of the S phase, with the induction of apoptosis. The AgNP (10 ppm) injected into fertilized eggs on days 5, 11 and 17 of incubation did not influence the development of embryos but decreased the number and size of lymph follicles in the bursa of Fabricius [45].

The PCV results indicated that the provision of AgNP did not influence the percentage of red blood cells (Fig. 2). On the other hand, it was reported that the oral administration of AgNP induced some changes in the red blood compartment, such as increased red blood cell count [46] and coagulation parameters [47].

The present results demonstrated age-related differences in immunoglobulin concentrations, which could probably be ascribed to the infection with *C. jejuni* on day 11 of age. Immunoglobulins are generally associated with immune resistance to extracellular bacteria and viruses or other foreign substances. Therefore, their levels might be most appropriate to study diseases caused by extracellular microorganisms [48]. Plasma immunoglobulin levels were measured following *C. jejuni* infection of chickens on days 15, 22 and 30 of age. Noticeable, significantly lower levels of IgG and IgM were observed in AgNP supplemented chickens (Fig. 3b-c). The decrease in the levels of IgG and IgM in AgNP birds might be due to the microbial colonization and provision of AgNP via drinking water. Importantly, the impaired T–cell function might be a reason for reduced IgG levels. AgNP might impair intestinal actively transported sugars, amino acids, trace elements, and vitamins, and deficiencies of these nutrients may decrease antibody formation. Similar observations showed decreased plasma IgG levels in chickens treated with 10 ppm and 20 ppm AgNP but not infected with *C. jejuni* [49]. Reduction of bursa and spleen weights may be correlated to the decreased IgG levels. Therefore, the expression of immunological effects in this study was assumed to be the result of AgNP impairment. It could be that lymphocyte production and self- or non-self-antigen selection against *C. jejuni* infection changes with age, and could also be influenced by AgNP. Our results suggested that AgNP can diminish the activity of humoral immunity of broiler chickens by decreasing the levels of immunoglobulins in plasma.

In the present study, *NF-kB* and *TNF-α* expression was determined in chicken liver tissue to evaluate whether or not AgNP could modify inflammation at the transcriptional level (Fig. 4). NF-κB and TNF-α expressions are important in *Campylobacter* colonization as *C. jejuni* primarily colonized the lower small intestine and caecum, where remarkable histopathologic and ultrastructural changes in the epithelium were noticed. In a novel rabbit model, the pathogen induced intestinal inflammation had increased levels of IFN-γ, TNF-α, IL-1β, IL-2, IL-6, IL-8, and IL-22 gene expression. In the acute phase, the bacteria induced a significant increase in the expression of the most pro-inflammatory genes [50]. The infection of human intestinal epithelium with *Campylobacter* results in activation of *NF-kB*, which is needed for the induction of pro-inflammatory genes [51]. We have recently observed that *TNF-α* mRNA expression was consistent with 50 ppm when AgNP were injected into chicken embryos after LPS stimulation during the 19th day [52]. Results concerning the mRNA expression of *NF-kB* were not in agreement with those of the chicken embryo liver [53]. We suppose that this could be due to the time of exposure, tissue specificity and route of administration of AgNP. However, our results confirm the pro-inflammatory activity of AgNP, which has been previously observed in chickens and mice [54, 55]. Furthermore, an increased inflammatory response of the AgNP group in comparison with the control group indicates an over-immunostimulation activity of AgNP. The ability of nanoparticles to freely move into local lymphoid tissues and trigger antigen-presenting cells might be responsible for their immunostimulatory activity.

The exact mechanism and reason how and where AgNP induce pro-inflammatory effects are not known, but it has been reported that they stimulate production of reactive oxygen species (ROS), and thereby modulate intracellular calcium concentrations, activate transcription factors, and induce cytokine production [56]. The toxicity of nanoparticles is manifested by inflammation resulting from oxidative stress [57–59]. Recently, various studies with AgNP have been published, demonstrating conflicting results that silver nanoparticles are toxic [44, 59–63] or non-toxic [64–66]. Furthermore, they could be pro-inflammatory [47, 67–69] or anti-inflammatory agents [27, 28, 65]. Importantly, particle size-dependent effects of AgNP were observed with respect to cellular uptake, pro-inflammatory response and changes at the proteome level [70]. The 20 nm AgNP elucidated a higher inflammatory response than the 200 nm particles in Caco-2/TC7 cells. It might be that bigger particles have different transport rates, reduced interaction with the cellular membranes and are better retained in the gastrointestinal epithelial mucous layer than smaller particles. Thus, smaller particles may cross the mucus layer and reach the cells.

An increase in mRNA expression of inflammatory mediators and low IgG and IgM levels could be due to the nanoparticle uptake triggering cellular effects, leading to inflammatory responses. However, the conflicting results might indicate that AgNP have multiple cellular targets that vary among different cell type. These results are attributed to several confounding factors such as pH [71], continuous oral administration of AgNP, [47, 68] or high concentration, [28] or even the availability of free radicals to induce oxidative stress and damage cells [58]. We propose that nanoparticles time of exposure, route of administration, particles size, aggregate formation, and altered bio-distribution in the form of rapid clearance owing to non-specific pathogen clearance from the systemic circulation could serve as aided factors*.* One possible cause for the AgNP dependent initiation of inflammation could be the fact that they enhance the production of ROS. These oxygen-derived free radicals may lead to mitochondrial dysfunction, increased gene expression of inflammatory cytokines (TNF-α) and activation of specific transcription factors (NF-kB). The absorption, distribution, metabolism, excretion, and toxicity of AgNP are largely dependent on their physicochemical properties and the surrounding environmental conditions. However, we were unable to find a mechanism involved in the pro-inflammatory pathway of AgNP in the present work and could not verify the presence of AgNP in the digestive organs.

## Conclusions

Orally-administered AgNP via the drinking water (50 ppm) had no effect on the intestinal colonization of *C. jejuni* following infection and did not influence the intestinal microbial profile of broiler chickens. However, AgNP reduced body weight gain, lowered concentrations of plasma immunoglobulins and upregulated mRNA expression of *TNF-α* and *NF-kB*, indicating toxicity and impaired immune response. Thus, the use of orally administered AgNP might be harmful to the chicken health.

## Additional files

Additional file 1: Table S1. Average daily water intake and the concentrations of silver nanoparticles (AgNP) when chickens were infected with *C. jejuni. (DOCX 13 kb)*

Additional file 2: Table S2. Antibacterial effect of silver nanoparticles (AgNP) against *C. jejuni* by using chicken intestinal organ culture model (CIOC-model)*.* AgNP concentrations 10 ppm, 20 ppm were provided via drinking water, 40 ppm and 80 ppm were supplemented in intestinal organ culture. Bacterial count was done by standard plate count method. (DOCX 12 kb)

Additional file 3: Figure S1. Effect of silver nanoparticles (AgNP) on *C. jejuni* at various concentrations, using broth microdilution method by microtiter plate. Bacterial density was measured by the plate counter. D20 to D70 ppm means diluted AgNP concentration from stock solution, C80 and C50 ppm means stock AgNP solution. Media, with bacteria were considered as negative and positive controls. The mean values of 3 repetitions. 1a) Bacterial growth (Optical density (OD)) difference = Average of bacteria - Average of media. 1b) Bacterial growth difference (%) = Bacterial growth difference / Average of bacteria. (DOCX 20 kb)

## Abbreviations

ADFI: Average daily feed intake
ADWG: Average daily weight gain
ADWI: Average daily water intake
AgNP: Silver nanoparticles
*C. jejuni*: *Camplylobacter jejuni*
CCDA: Charcoal cefoperazone deoxycholate agar
CIOC: Chicken intestinal organ culture
*E. coli*: *Escherichia coli*
ELISA: Enzyme-linked immunosorbent assay
FCR: Feed conversion ratio
HRP: Horseradish-peroxidase
Igs: Immunoglobulins
LAB: Lactic acid bacteria
MRS: de Man Rogosa Sharpe
PCV: Packed cell volume
RIN: RNA integrity number
ROS: Reactive oxygen species
RT: Room temperature
TEM: Transmission electron microscopy
TMB: 3,3,5,5-tetramethylbenzidine
TSC: Tryptose sulfite cycloserine
