# Supplementary material for: Influence of silver nanoparticles on growth and health of broiler chickens after infection with Campylobacter jejuni
Source: BMC Vet Res. 2018 Jan 2;14:1. doi: 10.1186/s12917-017-1323-x (PMC5748950; doi:10.1186/s12917-017-1323-x)
Supplement: Supplementary file 1 — Average daily water intake and the concentrations of silver nanoparticles (AgNP) when chickens were infected with C. jejuni. (DOCX 13 kb) [file 12917_2017_1323_MOESM1_ESM.docx]

| Table 1. Average daily water intake and AgNP concentrations when chickens were infected with *C.jejuni* | | |
| --- | --- | --- |
| AGE | ADWI(g/bird) | ADNPI(mg/bird) |
| 1-11d | 50.09 | 2.50 |
| 11-15d | 98.05 | 4.90 |
| 15-22d | 163.09 | 8.15 |
| 22-30d | 281.14 | 14.06 |
| 0-30d | 165.16 | 8.26 |
| *The estimation of AgNP which single bird received for a day. ADWI-Average daily water intake (g per bird), ANPI- Average daily AgNP intake (mg per bird). The data represent values are mean of 6 isolators each with 15 birds | | |
