# Supplementary material for: Influence of silver nanoparticles on growth and health of broiler chickens after infection with Campylobacter jejuni
Source: BMC Vet Res. 2018 Jan 2;14:1. doi: 10.1186/s12917-017-1323-x (PMC5748950; doi:10.1186/s12917-017-1323-x)
Supplement: Supplementary file 2 — Antibacterial effect of silver nanoparticles (AgNP) against C. jejuni by using chicken intestinal organ culture model (CIOC-model). AgNP concentrations 10 ppm, 20 ppm were provided via drinking water, 40 ppm and 80 ppm were supplemented in intestinal organ culture. Bacterial count was done by standard plate count method. (DOCX 12 kb) [file 12917_2017_1323_MOESM2_ESM.docx]

**Supplementary table 2**

| Table 2. Antibacterial effect of silver nanoparticles on male broiler chickens against *C.jejuni* by using chicken intestinal organ culture model (CIOC-model)*.* AgNPs concentrations 10 ppm, 20 ppm were provided via drinking water, 40 ppm and 80 ppm were supplemented in intestinal organ culture. Bacterial count was done by standard plate count method | | | | | |
| --- | --- | --- | --- | --- | --- |
| Sampling time points | control | 10ppm AgNPs | 20ppm AgNPs | 40ppm AgNPs | 80ppm  AgNPs |
| 1 | 7.67 | 7.58 | 7.51 |  |  |
| 2 | 8.0 | 7.72 | 7.82 |  |  |
| 3 | 7.89 | 7.73 | 7.80 |  |  |
| 4 | 8.28 |  |  | NC | NC |
| NC= No colonies, ppm= parts per million. Microbial growth Log CFU /mL and different sampling time and nanosilver concentrations. | | | | | |
