# Supplementary material for: Influence of silver nanoparticles on growth and health of broiler chickens after infection with Campylobacter jejuni
Source: BMC Vet Res. 2018 Jan 2;14:1. doi: 10.1186/s12917-017-1323-x (PMC5748950; doi:10.1186/s12917-017-1323-x)
Supplement: Supplementary file 3 — Effect of silver nanoparticles (AgNP) on C. jejuni at various concentrations, using broth microdilution method by microtiter plate. Bacterial density was measured by the plate counter. D20 to D70 ppm means diluted AgNP concentration from stock solution, C80 and C50 ppm means stock AgNP solution. Media, with bacteria were considered as negative and positive controls. The mean values of 3 repetitions. 1a) Bacterial growth (Optical density (OD)) difference = Average of bacteria - Average of media. 1b) Bacterial growth difference (%) = Bacterial growth difference / Average of bacteria. (DOCX 20 kb) [file 12917_2017_1323_MOESM3_ESM.docx]

**Supplementary Figure.** 1a

**Supplementary Figure.** 1b
